# The correlation between upper extremity musculoskeletal symptoms and joint kinematics, playing habits and hand span during playing among piano students

**DOI:** 10.1371/journal.pone.0208788

**Published:** 2018-12-19

**Authors:** Yael Kaufman-Cohen, Sigal Portnoy, Ran Sopher, Lital Mashiach, Lilach Baruch-Halaf, Navah Z. Ratzon

**Affiliations:** 1 Department of Occupational Therapy, Sackler Faculty of Medicine, Tel Aviv University, Tel Aviv, Israel; 2 Department of Engineering, Tel Aviv University, Tel Aviv, Israel; University of Ontario Institute of Technology, CANADA

## Abstract

**Objective:**

We aimed to investigate the correlations between Upper Extremity Musculoskeletal Symptoms (MSD) and joint kinematics while playing the piano, as well as correlations between MSD and psychosocial, professional and personal habits, and bio-demographic risk factors of piano students.

**Method:**

This cross-sectional study included 15 piano students. The research tools included 3D motion capture, anthropometric measurements, and questionnaires for obtaining data about MSD, psychological, and personal factors.

**Results:**

The piano students recruited for this study experienced a variety of MSD during the past 12 months, with a particularly high prevalence of neck pain (80%). Extreme wrist extension and/or elbow flexion while playing the piano also correlated with MSD. Additionally, this study identified correlations between MSD and hand span (r = -.69, p≤.004) and number of playing hours per week (r = .58, p≤.024).

**Conclusions:**

Anthropometric factors and playing patterns should be considered together with well-known MSD risk factors, like extreme and repetitive movements. However, considering each joint singularly might not be sufficient to prevent the development of MSD when instructing the piano player; accordingly, joint synchronization should also be considered.

## 1. Introduction

Non-specific muscle and tendon pain such as numbness and tingling in the neck, shoulder, arm, wrist and/or hand could be warning signs of current or impending Musculoskeletal Symptoms (MSD) [1–3]. These complaints might be attributed to an array of tendon disorders, tendon sheaths, and compression or entrapment of peripheral nerves in several areas [4–7]. MSDs are common among professional musicians. The virtuosity of musicians is studied extensively using neuroimaging techniques, e.g. functional magnetic resonance imaging (fMRI) and electroencephalography (EEG) [8,9]. However, much less academic attention is invested in studying musculoskeletal pain associated with playing. Review of the current literature shows a high prevalence of this phenomenon among professional musicians, varying from 60% to 87% in professional instrumental musicians [4–6,10–14], reported a point prevalence of 63% of musculoskeletal complaints among music academy students [13,14]. Among the reported risk factors leading to the development of MSD are biomechanical risk factors such as repetitive movements, playing in awkward positions, static load caused by holding the musical instrument or sitting in a certain position throughout the entire repertoire and anthropometric characteristics, e.g. weight of the instrument [4, 15–18].

Among all musicians, pianists are most commonly affected by MSD, with prevalence varying from 50% to 77% [7,19–22]. Studies report that as many as 87% of college piano students were injured before their enrollment in the university, suggesting that the majority of MSD began when these students were teenagers or children [6,23,24]. Furthermore, specific surveys revealed that 62% to 73% of piano students experienced MSD when playing at least one selected piano technique [25]. This high prevalence of MSD in piano students is attributed to some of the biomechanical factors, e.g. age, number of hours per week spent playing, at least one hour of continuative playing without breaks, sedentary life style, and acceptability of “No pain, no gain” criterion [26]. These risk factors may contribute to the incidence of MSD, including inefficient techniques that exert force on the wrists, fingers, and elbows when playing the piano [27].

Studies searching for alternative risk factors for MSD in pianists, studied various characteristics such as the brand and model of the instrument, its year of construction, vertical versus coda instrument and anthropometrics [18,28]. These alternate factors may be important because the joint kinematics may change when using different pianos [27]. Despite these variations, only a few studies have examined the relation between MSD and joint kinematics [13,29,30].

Related literature is scarce and needed in order to understand the movement characteristics that enable extremely fast cyclic limb motion during musical performance and its correlation to MSD. Therefore, we aimed to investigate (i) the correlations between MSD and joint kinematics during piano playing, and (ii) the correlations between MSD and psychosocial, professional and personal habits, and bio-demographic risk factors in piano students. We hypothesized that relationships will be registered between MSD and joint kinematics while playing the piano and between MSD and several other risk factors that musicians are known to be exposed to (such as and psychosocial, professional and personal habits, and bio-demographic risk factors).

## 2. Methods

This was a cross-sectional study in which piano students filled out a questionnaire regarding MSD and the kinematic patterns of their piano playing were recorded. The study was approved by the Institutional Ethics Committee at Tel Aviv University.

### 2.1 Participants

We recruited 15 piano students (nine males and six females) by convenience and snowball sampling methods, from the school of music at Tel-Aviv University, with a mean and Standard Deviation (SD) of age 21.72.4± years (range 19–27 years). Three participants were left-handed. Inclusion criteria required that students had at least five years of experience playing the piano and were studying for their bachelor degree. Exclusion criteria were limitations of range of motion due to an orthopedic or a neurologic diagnosis.

### 2.2 Research tools

We incorporated the use of questionnaires and a motion capture system, as detailed below.

#### 2.2.1 Questionnaires

We used the Standardised Nordic Questionnaires (SNQ) [31], which screens musculoskeletal pain in an ergonomic or occupational health context. The questionnaire includes a detailed reference to pain in nine different parts of the body (neck, shoulder, elbow, wrist, lower and upper back, hip, knee, and ankle) during the past week and the last year. Consequently, there were 5 different variables concerning musculoskeletal symptoms, explaining the prevalence of Musculoskeletal Disorders (MSD): 1. Number of symptomatic musculoskeletal joints, in the past year, 2. Number of symptomatic musculoskeletal joints, in the past week, 3. Number of symptomatic upper limb musculoskeletal joints, in the past year, 4. Number of symptomatic upper limb musculoskeletal joints, in the past week, 5. Work limitations due to symptomatic body regions, in the past year.

The scale does not denote severity only presence/absence of pain. The SNQ has been shown to be both reliable and valid when compared with clinical evaluation [32].

In this study, we used the SNQ and a validated appendix for Upper Extremities (UE) that uses the same format of the SNQ (mapping painful joints in the musculoskeletal system and their locations). This addition focused on reviewing the presence of pain in the muscles of the arm, elbow, forearm, palm and each of the fingers [33].

Finally, we used a personal questionnaire that included information regarding professional playing habits.

#### 2.2.2 Joint kinematics

A six-camera high-speed motion analysis system (Qualysis Medical AB, Sweden) was used to capture the movement of the subjects while playing a piano. One camera was set on the ceiling, above the piano keys. The rest of the cameras were set on tripods around the subject, and set so that each marker was visible to at least two cameras at all times. The system calibration was performed according to the guidelines of the manufacturer. Hand and wrist movements were derived from tracking 11 small (7mm in diameter) passive reflective markers adhered to the skin of the subject on anatomical landmarks of the right hand and arm (3 markers on the torso, 3 on the upper arm, 3 on the forearm, and 2 on the hand). Marker placement allowed computing of the elbow and wrist angles, i.e. elbow flexion and extension, ulnar and radial wrist deviation, and wrist flexion and extension. Data were collected at 100 Hz.

### 2.3 Study protocol

Each subject read and signed an informed consent form. After filling out the three aforementioned questionnaires, anthropometric measurements of the subjects were taken, including lengths of the limb, arm, forearm, third digit, and hand span by measuring the distance between the tips of the thumb and the small finger [34]. The subjects were instructed to play a standard-sized piano, which was placed in the motion laboratory. The height of the piano keyboard was 71cm and the subjects sat on a piano stool, adjusted to a comfortable height.

The subjects played the piano for two minutes for warming up. Then the subjects were asked to sit with the elbow flexed to 90° and the pronated forearm for a static trial. The subjects then played a musical piece (Praeludium I, in C, by Bach) recurrently for 10 minutes, recommended by the head of the piano department as a basic piece, known and practiced by all students. Data were recorded within a 10-minute session at three one-minute intervals: at the third, sixth, and ninth minutes.

### 2.4 Data analysis

The percentage of subjects who experienced pain was calculated. The SNQ and its Appendix scores were calculated and analyzed. The most accurate one-minute recording, in terms of marker tracking, was selected for the analysis. The 3D coordinates of the markers were filtered using a 7^th^-order Butterworth low pass filter with a cut-off frequency of 5Hz and the joint angles, velocities, and accelerations were calculated in MatLAB, version 7.9. We calculated the coefficient of correlation between elbow and wrist flexion-extension angles, as a measure of joint synchronization. In addition, we calculated the correlation between elbow flexion-extension of the elbow and wrist flexion extension separately for each subject, using the values retrieved while sampling ROM of playing.

Correlation tests were used in two stages. First, the Spearman Rank Correlation test was used to test the correlation between frequency of MSD and bio-demographic variables. We found that MSD of the hand correlated with hand span and MSD of the body correlated with the number of practicing hours during the week. Following this finding, we performed independent analyses of the correlation between the results of the kinematic data and MSD of the hand (controlling for hand span) versus the MSD of the body (controlling for the number of practicing hours during the week). A Spearman Rank Correlation test was used to test the correlation between the kinematic data and MSD interfering with Activities of Daily Living (ADL) in the preceding year. An alpha level below 0.05 was considered significant. Statistical analyses were performed with SPSS version 18.

## 3. Results

### 3.1 Prevalence of pain, bio-demographic data and playing factors

Table 1 depicts the prevalence of body parts experiencing pain in the preceding year, in the preceding week, and MSD interfering with ADLs in the preceding year. The piano students experienced a variety of MSD in the preceding year, with a particularly high prevalence of neck pain (80%), upper back pain (60%), and lower back pain (53.3%).

**Table 1:** The prevalence of body parts experiencing pain in the preceding year, in the preceding week (presented my numbers and percentage), and Musculoskeletal Disorders (MSD) interfering with Activities of Daily Living (ADL) in the preceding year (N = 15). For body parts where no pain was reported, data are not shown.

Body part		Painful body parts in preceding year		Painful body parts in preceding week^1^		MSD of the body interfering with ADL in preceding year^1^	
		Number	Percent	Number	Percent	Number	Percent
Neck		12	80	7	46.7	2	13.3
Shoulder	Right	2	13.3	-	-	-	-
	Left	1	6.7	-	-	-	-
	Both	5	33.3	7	46.7	1	6.7
Elbow	Right	1	6.7	-	-	-	-
	Left	0	0	-	-	-	-
	Both	3	20	2	13.3	1	6.7
	Right	4	22.2	-	-	-	-
Arm	Left	4	22.2	-	-	-	-
	Both	-	-	4	22.2	2	11.1
Forearm	Right	6	33.3	-	-	-	-
	Left	4	22.2	-	-	-	-
	Both	-	-	7	38.9	2	11.1
Wrist	Right	1	6.7	-	-	-	-
	Left	1	6.7	-	-	-	-
	Both	5	33.3	5	33.3	1	6.7
	Right	2	11.1	-	-	-	-
Palm	Left	4	22.2	-	-	-	-
	Both	-	-	4	22.2	2	11.1
	Right	1	5.6	-	-	-	-
Thumb	Left	1	5.6	-	-	-	-
	Both	-	-	2	11.1	-	-
2^nd^ finger	Right	1	5.6	-	-	-	-
	Both	-	-	1	5.6	3	16.7
3^rd^ finger	Right	1	5.6	-	-	-	-
	Both	-	-	2	11.1	3	16.7
4^th^ finger	Right	1	5.6	1	5.6	3	16.7
	Left	1	5.6	-	-	-	-
5^th^ finger	Right	2	11.1	1	5.6	-	-
Back	Upper	9	60	6	40	2	13.3
	Lower	8	53.3	3	20	1	6.7
Hip		3	20	2	13.3	-	-
Knee		2	13.3	1	6.7	1	6.7
Ankle		2	13.3	1	6.7	1	6.7

^1^The original questionnaire does not differentiate between sides, but refers to one or two sides.

Mean and SD of Body Mass Index (BMI) and professional habits of the study population are presented in Table 2. The subjects differed from one another in their playing habits. All had at least six years of playing experience and played an average of more than three hours per day.

**Table 2:** Bio-demographic variables, habits of study population, and kinematic data while playing the piano (N = 15).

			Minimum	Maximum	Mean	SD
Kinematic data		Elbow flexion-extension (°)	29.1	103.9	73.5	13.1
		Wrist flexion-extension (°)	-31.2Flex	37.5Ext	-5.2Flex	11.0
		Wrist ulnar-radial deviation (°)	-7.7RD	11.1UD	4.5UD	6.5
Body Mass Index (BMI)			16.9	23.4	19.6	1.9
Anthrop. data	Limb Length (cm)		65.0	87.0	72.5	5.2
	Arm Length (cm)		26.0	34.0	28.5	2.1
	Forearm Length (cm)		24.0	32.0	27.0	2.5
	3^rd^ Finger Length (cm)		15.0	19.0	16.6	1.1
	Hand Span (cm)		19.0	26.5	21.3	2.1
Playing Habits	Playing Piano (years)		6.0	20.0	13.4	3.5
	Playing Piano (hours per week)		10.5	39.0	23.9	8.1
	Body Warming (min.)		0.0	45.0	8.1	13.8
	Break Duration (min.)		0.0	45.0	15.2	14.3
Personal Habits	Physical Training (hours per week)		0.0	5.5	1.5	2.0
	Housework (hours per week)		0.0	6.0	1.3	1.9

SD = standard deviation; Antrop = Antropometric

*Maximal elbow extension is defined as 0°

Flex = Flexion; Ext = Extension; RD = radial deviation; UD = ulnar deviation

### 3.2 Joint kinematics

Kinematic data obtained during piano playing are presented in Table 2. Kinematic data acquired while the subjects played the piano mostly showed flexion at the elbow joint with the wrist in an almost neutral position, favoring a slight ulnar deviation.

**Fig 1:**
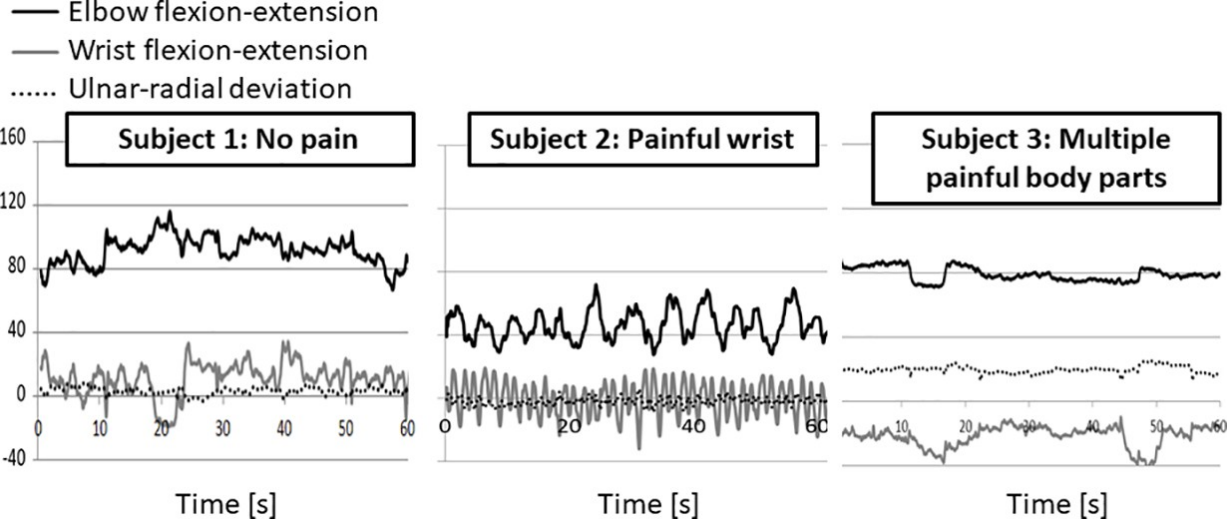
Example of the kinematic data of 3 subjects playing the piano for duration of 1 minute. Elbow flexion is below 90° and elbow extension is above 90°. Wrist flexion and radial deviation are negative values. Wrist extension and ulnar deviation are positive values.

Examples of the kinematic data of three subjects playing the piano for a one-minute duration is depicted in Fig 1. In these examples, Subject 3, who complained of pain in multiple body parts, demonstrated a restricted range of motion in both joints. Subject 3 played with a flexed wrist for the entire minute of recorded time, whilst Subjects 1 and 2 demonstrated both flexion and extension of the wrist, and displayed a more dynamic and less fluctuating movement strategy than Subject 3.

### 3.3 Correlations

The results for the first study aim, correlation analyses are shown in Table 3. Most correlations were found between MSD and the extreme wrist and elbow joint angles recorded while playing the piano. We found no correlation between the MSD and the coefficient of correlation between elbow and wrist flexion-extension. Yet when, the correlation between elbow flexion-extension of the elbow and wrist flexion extension was calculated separately for each subject we found significant correlation between elbow and wrist sagittal motion in 14 out of 15 subjects (the range of Pearson's coefficient of correlation was -0.03 to -0.65). The negative correlation indicates that when the elbow extends, the wrist flexes. Since no significant correlations were demonstrated between other kinematic variables (e.g. velocity, acceleration) and MSD, data are not shown.

**Table 3:** The correlation between maximal or minimal joint angles and Musculoskeletal Disorders (MSD).

	MSD of the hand during the preceding week		MSD of the hand during the preceding year		MSD of the body during the preceding week		MSD of the body during the preceding year		MSD interfering during the preceding year	
	r	p	r	p	r	p	r	p	r	p
Max. Elbow angle**	-.19	.51	.17	.55	.22	.46	.15	.60	.09	.75
Min. Elbow angle^¥^	.21	.46	.41	.15	.53*	.04	.46	.10	.61*	.01
Max. Wrist angle**	.57*	.03	.43	.12	.67*	.01	.61*	.02	.54*	.04
Min. Wrist angle^¥^	.41	.14	.31	.27	.72*	.004	.16	.57	.16	.56
Max. RUD^§^	-.25	.38	-.03	.91	-.32	.27	-.42	.13	.-55*	.03
Min. RUD^Ψ^	-.27	.35	-.08	.77	-.08	.78	-.09	.75	.39	.15

*P<0.05

** toward extension

¥ toward flexion

§ toward ulnar deviation

Ψ toward radial deviation

Max = maximal; Min = Minimal; RUD = Radial-Ulnar Deviation.

Two variables were associated with MSD: hand span correlated with MSD of the hand experienced during the preceding week (r = -.69, p≤.004) and the preceding year (r = -.56, p≤.023). The results for the second study aim showed that no correlations were found between MSD and risk factors in piano students except for one correlation between the number of playing hours per week and MSDs of the body during the preceding week (r = .58, r≤.024).

## 4. Discussion

The aim of this cross-sectional study was to investigate the correlations between the frequency of MSD and joint kinematics during piano playing. Professional habits of piano students were correlated with MSD. As discussed below, this study identified correlations between MSD and the joint angles of the excessive wrist and elbow recorded while playing the piano. Additionally, MSD were found to correlate with hand span and with the number of practice hours playing per week.

Instrumental musicians in general and pianists in particular, are at a risk group for injuries resulting from repetitive motion. The relatively high prevalence of MSD that we found in this study is similar to previous published outcomes for piano students [25,35]. Since playing an instrument will usually affect the UE and the neck regions, it is conceivable that musicians have more complaints related to the upper body than non-musicians [13]. The high prevalence of MSD may also be explained by the fact that musicians take up their instruments at an early age, thus exposing them to soft tissue injury [36].

Sugawara, [37] in his study on musician's wrist postures reported that pianists’ wrists are typically noticed to be in 15° of ulnar deviation and slight extension [38]. Ulnar deviation past 20° and 15° of wrist extension may cause an increased pressure in the carpal tunnel [39]. When observing the elbow, Fillion [40], reported that pianists sit in front of the piano with their elbows flexed approximately 90° and with their wrists close to the neutral position.

The kinematics recorded for wrist and elbow angles in this study are also similar to the published literature [37]. We found comprehensive and statistically significant association between wrist and elbow kinematics and the experience of MSD amongst piano students during the preceding week and preceding year (Table 3). In this study most correlations were found between the wrist angles (mostly toward extension) and MSD of the hand and body, both in the preceding week and the preceding year, and leading to work limitations due to symptomatic body regions, in the past year. These findings reinforce previous literature, which showed that awkward movements may provoke musculoskeletal pain, especially in the wrists and hands [41]. Another explanation was suggested by [42], who found that extended wrist position (at ~20°) significantly increased the force produced by the fingers. The increase in striking force might result in an increase in joint force, thereby contributing to joint pain. The MSD experienced by subjects with excessive wrist extension might also be explained by compensatory movement of proximal joints, e.g. the shoulders. During wrist extension when the hand is positioned near the body, i.e. shoulder adduction, the shoulders are prone to be elevated. Due to the mechanical interaction between linked segments, a joint motion originates not only from the torque generated by its surrounding muscles but also from torque generated at the adjacent joints [43]. Therefore, muscular torque should compensate for these inter-segmental dynamics in order to generate a planned motion [44]. Moreover, this compensatory muscular work increases at a faster rhythm in repetitive arm movements [45], e.g. during piano playing. An interesting finding of this study was the negative correlation between interferences in ADLs due to body pain in the previous year and ulnar deviation in the wrist. It is expected that playing with excessive ulnar deviation would cause uncomfortable wrist pain that might affect ADL [37]. Our findings might not contradict this expectation, since the mean (and SD) of the ulnar deviation of our subjects was 5.6±7.0°, a range that is not considered extreme and therefore might not cause MSD that can affect ADL (Table 2).

Excessive elbow angles (toward flexion) correlated significantly with musculoskeletal pain in the total body during the preceding week. This kind of correlation was found also among typists, who likewise demonstrate a significant correlation between elbow angles and musculoskeletal pain of different regions of the body [46]. These findings could be explained through compensatory body movements [27] incorporated while playing the instrument. Excessive joint angles in the wrist and elbow (toward extension in the wrist and flexion in the elbow; Table 3) mostly correlated with MSD experienced in the preceding week. Expectedly, these joint angles also correlated with MSD interfering in ADL in the past year (Table 3).

The maximal and minimal joint angles might not be enough to characterize the playing patterns of piano students. It is also important to observe the synchronization between the joints. Although we did not find correlations between the MSD and the coefficient of correlation between elbow and wrist flexion-extension, it appears that referring to the range of motion of the elbow and wrist each by itself is insufficient. We found negative correlations between wrist and elbow movement in the sagittal plane for most subjects (93%). This finding emphasizes the importance of considering the joint synchronization of the piano player when the ergonomist instructs him or her toward safer playing strategies, specifically for students developing new playing habits. In Fig 1, we present three cases in which maximal joint angles do not correlate with their reported MSD. Subject 1 reported no joint pain. This subject exhibited fluctuating movement in the elbow and wrist, repeatedly changing from flexion to extension and so forth. This subject also maintained a wrist angle of between 0° and 10° toward ulnar deviation. Although Subject 1 extended the wrist to almost 40°, the subject reported no pain. Subject 2 reported pain in his wrists, but did not extend the wrist by more than 20°. This subject exhibited extreme elbow flexion throughout the entire minute of recording. Like Subject 1, Subject 2 alternated between flexion and extension of the wrist; unlike the first subject, Subject 2 did so with extreme frequency. Although the range of motion of the wrist for Subjects 1 and 2 was similar, their movement pattern was different from one another. Like Subject 1, Subject 2 also maintained a wrist angle of 0° to 10° toward ulnar deviation. Subject 3 experienced pain in multiple body parts. This subject maintained a wrist flexion of approximately 20° of ulnar deviation. During the minute of recording, Subject 3 kept a stiff posture of both elbow and wrist compared with Subjects 1 and 2. This reported body parts experiencing pain of this subject could be explained by compensatory movement of the shoulder and torso, the latter affecting neck pain. Aspects of motor control and learning, precision, timing, strategies for compensation, adaptation and coordination can be observed during playing [47]. Studies have demonstrated that individuals with superior piano skills economize the work performed by distal muscles during a piano keystroke by taking advantage of proximal joint motion [29].

This study also identified a correlation between MSD and hand span. A larger hand span enables certain playing techniques, such as simultaneous playing of the thumb and fourth or fifth fingers. Playing an octave accounts for 74% of the piano techniques that pianists practice [34]. We found significant correlation between hand span and MSD of the hand during the preceding week and preceding year. This finding supports similar outcomes of previous research reporting that pianists with small hands are prone to overuse injuries in the upper extremities [21].

Finally, this study identified a correlation between the number of playing hours per week and MSD of the body during the preceding week. This outcome reinforces previous research findings of positive correlations between the average of playing hours in an orchestra and playing-related musculoskeletal complaints [18,48] Conversely, in a cross-sectional study among piano teachers, playing time was inversely related with musculoskeletal complaints [49]. To date, no longitudinal cohort study has been performed among professional or student musicians. There are studies proving that paying minimal attention to warming up prior to playing or practicing, the duration of play, and taking insufficient breaks, ultimately provokes MSD [21]. For example, piano students who practiced more than 20 hours per week were found to have a higher incidence of MSD [50]. Several studies of young adults found an association between increased practice time and increased pain [38,51]. An abrupt increase in practice time is the most important risk factor for MSD [4]. Evidence suggests that musicians should take frequent rest breaks and practice for shorter periods of time to prevent music related injuries [52]. Further extensive work is needed, with a larger number of subjects, in order to reach an unequivocal conclusion on the correlation between upper extremity MSD and joint kinematics, playing habits and hand span during playing among piano students.

The main limitation of this study is that joints other than the elbow and wrist were not monitored, so that the compensation techniques are not recorded. Similarly, little attention was payed to control the differences of participants’ height and its impact on kinematics. In addition, not having all participants playing in the same tempo like when following a metronome could have some effect on the playing fragment or hand positions, muscle tension and so on. Another limitation of the study is the recruitment procedure that might bias the results due to the homogeneity of the selected population Also, the small sample size of participants might not be representative of the population of student pianists. Finally, this cross-sectional study does not track the development of pain over time.

## 5. Conclusions

We found that extreme wrist extension and/or elbow flexion while playing the piano correlated with MSD. However, considering each joint individually might not be sufficient when instructing the piano player; joint synchronization should also be considered. Also, we found correlations between MSD and hand span and between MSD and number of playing hours per week. We conclude that these factors should be considered together with well-known MSD risk factors like extreme and repetitive movements. Piano students should adopt healthy playing habits, e.g. incorporating fewer hours of practice per day or decrease extreme wrist extension (or longer breaks and worm ups).

Further longitudinal research should seek to identify causal factors for MSD and should examine the role of synchronization between different anatomical joints as risk factors for MSD. Such research should include a larger sample size of participants and a range of ages, beginning with very young students, in order to determine early health education programs for instructing students in appropriate kinematics.

## Supporting information

S1 File Data set. (SAV)

## References

[1] Kennedy CA, Amick BC III, Dennerlein JT, Brewer S, Catli S, Williams R, et al Systematic review of the role of occupational health and safety interventions in the prevention of upper extremity musculoskeletal symptoms, signs, disorders, injuries, claims and lost time. Journal of occupational rehabilitation 2010;20(2):127–162. 10.1007/s10926-009-9211-2 19885644

[2] Hagberg M. Work Related Musculoskletal Disorders (WMSDs). A reference book for prevention. 1995;115–137.

[3] Gold JE, Hallman DM, Hellström F, Björklund M, Crenshaw AG, Djupsjobacka M, et al Systematic review of biochemical biomarkers for neck and upper-extremity musculoskeletal disorders. Scandinavian Journal of Work, Environment and Health 2016;42(2):103–124. 10.5271/sjweh.3533 26599377

[4] Hoppmann RA. Instrumental musicians' hazards. Occupational Medicine (Philadelphia, Pa.) 2001; 6(4):619–31 iv-v. 11567922

[5] Fry HJ. Incidence of overuse syndrome in the symphony orchestra. Medical Problems of Performing Artists 1986;1:51–55.

[6] Bragge P, Bialocerkowski A, McMeeken J. A systematic review of prevalence and risk factors associated with playing-related musculoskeletal disorders in pianists. Occupational Medicine 2006;56(1):28–38. 10.1093/occmed/kqi177 16275655

[7] Zaza C, Charles C, Muszynski A. The meaning of playing-related musculoskeletal disorders to classical musicians. Social Science & Medicine 1998;47(12):2013–2023. 10075243 10.1016/s0277-9536(98)00307-4

[8] Wan CY, Schlaug G. Music making as a tool for promoting brain plasticity across the life span. Neuroscientist 2010;16(5):566–577. 10.1177/1073858410377805 20889966 PMC2996135

[9] Pantev C, Herholz SC. Plasticity of the human auditory cortex related to musical training. Neuroscience & Biobehavioral Reviews 2011;35(10):2140–2154. 21763342 10.1016/j.neubiorev.2011.06.010

[10] Silva AG, Lã FM, Afreixo V. Pain prevalence in instrumental musicians: A systematic review. Medical Problems of Performing Artists 2015;30(1):8 10.21091/mppa.2015.1002 25743601

[11] Kaneko Y, Lianza S, Dawson WJ. Pain as an incapacitating factor in symphony orchestra musicians in Sao Paulo. Brazil. Medical Problems of Performing Artists 2005;20(4):168–174.

[12] Engquist K, Orbaek P, Jakobsson K. Musculoskeletal pain and impact on performance in orchestra musicians and actors. Medical Problems of Performing Artists 2004;19(2):55–61.

[13] Kok LM, Vlieland TP, Fiocco M, Nelissen RG. A comparative study on the prevalence of musculoskeletal complaints among musicians and non-musicians. BMC Musculoskeletal Diseases 2013a;14:9. 10.1186/1471-2474-14-9 PMC3554565 23289849

[14] Kok LM, Vliet Vlieland TP, Fiocco M, Kaptein AA, Nelissen RG Musicians’ illness perceptions of musculoskeletal complaints. Clinical Rheumatology 2013b;32(4):487–492. 23417426 10.1007/s10067-013-2199-1

[15] Malchaire J, Roquelaure Y, Cock N, Piette A, Vergracht S, Chiron H. Musculoskeletal complaints, functional capacity, personality and psychosocial factors. International Archives of Occupational and Environmental Health 2001;74(8):549–557. 11768043 10.1007/s004200100264

[16] Brandfonbrener AG. Musculoskeletal problems of instrumental musicians. Hand Clinics 2003;19(2):231–9. 12852665 10.1016/s0749-0712(02)00100-2

[17] Hansen PA, Reed K. Common musculoskeletal problems in the performing artist. Physical Medicine and Rehabilitation Clinics 2006;17(4):789–801. 10.1016/j.pmr.2006.08.001 17097480

[18] Kaufman-Cohen Y, Ratzon N. Correlation between risk factors and musculoskeletal disorders among classical musicians. Occupational Medicine 2011;61(2):90–95. 10.1093/occmed/kqq196 21273187

[19] Pak CH, Chesky K. Prevalence of hand, finger, and wrist musculoskeletal problems in keyboard instrumentalists. Medical Problems of Performing Artists 2001;16(1):17–23.

[20] Yoshimura E, Paul PM, Aerts C, Chesky K. Risk factors for piano-related pain among college students. Medical Problems of Performing Artists 2006;21:118–125.

[21] Furuya S, Nakahara H, Aoki T, Kinoshita H. Prevalence and causal factors of playing related musculoskeletal disorders of the upper extremity and trunk among Japanese pianists and piano students. Medical Problems of Performing Artists 2006;21(3):112–117.

[22] Abréu-Ramos AM, Micheo WF. Lifetime prevalence of upper-body musculoskeletal problems in a professional-level symphony orchestra: age, gender, and instrument-specific results. Medical Problems of Performing Artists 2007;22(3):97.

[23] Warrington J, Winspur I, Steinwede D. Upper-extremity problems in musicians related to age. Medical Problems of Performing Artists 2002;17(3):131–134.

[24] Brandfonbrener AG. History of playing-related pain in 330 university freshman music students. Medical Problems of Performing Artists 2009;24(1):30.

[25] Dockrell NSS. The prevalence of injuries among pianists in music schools in Ireland. Medical Problems of Performing Artists 2000;15(4):155.

[26] Bruno S. Lorusso A. L’Abbate N. Playing-related disabling musculoskeletal disorders in young and adult classical piano students. International Archives of Occupational and Environmental Health 2008;81(7):855–860. 10.1007/s00420-007-0279-8 18210148

[27] Allsop L, Ackland T. The prevalence of playing-related musculoskeletal disorders in relation to piano players' playing techniques and practicing strategies. Music Performance Research 2010;3(1):61–78.

[28] Wagner C. The pianist's hand: anthropometry and biomechanics. Ergonomics 1988;31(1):97–131. 10.1080/00140138808966651 3359991

[29] Furuya S. Goda T. Katayose H. Miwa H. Nagata N. Distinct inter-joint coordination during fast alternate keystrokes in pianists with superior skill. Frontiers in Human Neuroscience 2011;5:50–52. 10.3389/fnhum.2011.00050 21660290 PMC3107480

[30] Dalla Bella S. Palmer C. Rate effects on timing, key velocity, and finger kinematics in piano performance. PloS One 2011;6(6):e20518 10.1371/journal.pone.0020518 21731615 PMC3121738

[31] Kuorinka I, Jonsson B, Kilbom A, Vinterberg H, Biering-Sørensen F, Andersson G, et al Standardised Nordic questionnaires for the analysis of musculoskeletal symptoms. Applied Ergonomics 1987;18(3):233–237. 15676628 10.1016/0003-6870(87)90010-x

[32] Palmer K, Smith G, Kellingray S, Cooper C. Repeatability and validity of an upper limb and neck discomfort questionnaire: the utility of the standardized Nordic questionnaire. Occupational Medicine 1999;49(3):171–175. 10451598 10.1093/occmed/49.3.171

[33] Ratzon N, Mizrachi N. The presence of musculoskeletal disorders among amateur bowlers. Work: A Journal of Prevention, Assessment and Rehabilitation 2008;30(4):369–375. 18725700

[34] Sakai N, Liu MC, Su FC, Bishop AT, An KN. Hand span and digital motion on the keyboard: concerns of overuse syndrome in musicians. The Journal of hand surgery 2006;31(5):830–835. 10.1016/j.jhsa.2006.02.009 16713851

[35] Corrêa LA, dos Santos LT, Paranhos ENN, Albertini AIM, Parreira PDCS, Nogueira LAC. Prevalence and Risk Factors for Musculoskeletal Pain in Keyboard Musicians: A Systematic Review. PM&R 2018;10(9):942–950. 29705171 10.1016/j.pmrj.2018.04.001

[36] Rosety-Rodriguez M, Ordóñez F, Farias J, Rosety M, Carrasco C, Ribelles A, et al The influence of the active range of movement of pianists' wrists on repetitive strain injury. European Journal of Anatomy 2013;7(2):75–77.

[37] Sugawara E. The study of wrist postures of musicians using the Wrist System (Greenleaf Medical System). Work: A Journal of Prevention, Assessment and Rehabilitation 1999;13(3):217–228. 12441547

[38] Tubiana R, Chamagne P, Brockman R. Fundamental positions for instrumental musicians (third of a series of three articles). Medical Problems of Performing Artists 1989;4(2):73.

[39] Hagglund KL, Jacobs K. Physical and mental practices of music students as they relate to the occurrence of music-related injuries. Work: A Journal of Prevention, Assessment and Rehabilitation 1996;6(1):11–24. 10.3233/WOR-1996-6103 24441426

[40] Fillion P. Proximal Stability: A factor in musicians’ upper extremity pain. Work: A Journal of Prevention, Assessment and Rehabilitation 1999;2: 57–60.

[41] Brandfonbrener AG. The epidemiology and prevention of hand and wrist injuries in performing artists. Hand Clinics 1990 8;6(3):365–377. 2211849

[42] Li Z. The influence of wrist position on individual finger forces during forceful grip. Journal of Hand Surgery-American Volume 2002;27(5):886–896. 10.1053/jhsu.2002.35078 12239681

[43] Dounskaia N. Control of human limb movements: the leading joint hypothesis and its practical applications. Exercise and Sport Sciences Reviews 2010;38(4):201 10.1097/JES.0b013e3181f45194 20871237 PMC2965031

[44] Gribble PL, Ostry DJ. Compensation for interaction torques during single-and multijoint limb movement. Journal of Neurophysiology 1999;82(5):2310–2326. 10.1152/jn.1999.82.5.2310 10561408

[45] Dounskaia N, Swinnen S, Walter C, Spaepen A, Verschueren S. Hierarchical control of different elbow-wrist coordination patterns. Experimental Brain Research 1998;121(3):239–254. 9746130 10.1007/s002210050457

[46] Levanon Y, Gefen A, Lerman Y, Givon U, Ratzon NZ. Reducing musculoskeletal disorders among computer operators: comparison between ergonomics interventions at the workplace. Ergonomics 2012;55(12):1571–1585. 10.1080/00140139.2012.726654 23039764

[47] Metcalf CD. Irvine TA. Sims L. Wang YL. Su AWY. Norris DO. Complex hand dexterity: a review of biomechanical methods for measuring musical performance. Frontiers in Psychology 2014;5:414–426. 10.3389/fpsyg.2014.00414 24860531 PMC4026728

[48] Ackermann B. Driscoll T. Kenny DT. Musculoskeletal pain and injury in professional orchestral musicians in Australia. Medical Problems of Performing Artists 2012; 27(4):181–187. 23247873

[49] Yoshimura E. Fjellman-Wiklund A. Paul PM. Aerts C. Chesky K. Risk Factors for Playing-related Pain among Piano Teachers. Medical Problems of Performing Artists 2008;23(3):107–113.

[50] Foxman I, Burgel BJ. Musician health and safety: Preventing playing-related musculoskeletal disorders. AAOHN J 2006 7;54(7):309–316. 10.1177/216507990605400703 16862878

[51] Fjellman-Wiklund A, Sundelin G. Musculoskeletal discomfort of music teachers: an eight-year perspective and psychosocial work factors. International Journal of Occupational and Environmental Health 1998;4(2):89–98. 10.1179/oeh.1998.4.2.89 10026470

[52] Kok LM. Huisstede BM. Douglas TJ. Nelissen RG. Association of Arm Position and Playing Time with Prevalence of Complaints of the Arm, Neck, and/or Shoulder (CANS) in Amateur Musicians: A Cross-Sectional Pilot Study Among University Students. Medical Problems of Performing Artists 2017;32(1):8–12. 10.21091/mppa.2017.1003 28282473

